# Relapsing tricuspid valve endocarditis by multidrug-resistant *Pseudomonas aeruginosa* in 11 years: tricuspid valve replacement with an aortic valve homograft

**DOI:** 10.1186/s13019-015-0287-9

**Published:** 2015-06-09

**Authors:** Min-Seok Kim, Hyoung Woo Chang, Seung-Pyo Lee, Dong Ki Kang, Eui-Chong Kim, Ki-Bong Kim

**Affiliations:** 1 Department of Thoracic and Cardiovascular Surgery, Seoul National University Hospital, 101 Daehak-ro, Jongno-gu, Seoul 110-744 Korea; 2 Department of Internal Medicine, Seoul National University Hospital, 101 Daehak-ro, Jongno-gu, Seoul 110-744 Korea; 3 Department of Laboratory Medicine, Seoul National University Hospital, 101 Daehak-ro, Jongno-gu, Seoul 110-744 Korea

**Keywords:** Endocarditis, Prosthesis, Tricuspid valve surgery

## Abstract

Eleven years ago, a 27-year-old non-drug abuser woman was admitted to the hospital due to a burn injury. During the treatment, she was diagnosed with tricuspid valve infective endocarditis caused by multi-drug resistant (MDR) *Pseudomonas aeruginosa* (*P. aeruginosa*). She underwent tricuspid valve replacement (TVR) using a bioprosthetic valve, followed by 6 weeks of meropenem antibiotic therapy. Ten years later, she was again diagnosed with prosthetic valve infective endocarditis caused by MDR *P. aeruginosa*. She underwent redo-TVR with a bioprosthetic valve and was treated with colistin and ciprofloxacin. Ten months later, she was again diagnosed with prosthetic valve infective endocarditis with MDR *P. aeruginosa* as a pathogen. She underwent a second redo-TVR with a tissue valve and was treated with colistin. Two months later, her fever recurred and she was again diagnosed with prosthetic valve infective endocarditis caused by MDR *P. aeruginosa*. She eventually underwent a third redo-TVR using an aortic valve homograft and was discharged from the hospital after additional 6 weeks’ of antibiotic therapy. All the strains of *P. aeruginosa* isolated from each event of infective endocarditis were analyzed by repetitive deoxyribonucleic acid sequence-based polymerase chain reaction (rep-PCR) deoxyribonucleic acid (DNA) strain typing to determine the correlation of isolates. All of the pathogens in 11 years were similar enough to be classified as the same strain, and this is the first case report of TVR using an aortic valve homograft to treat relapsing endocarditis.

## Background

Repeat infective endocarditis due to the same species is traditionally defined as a “relapse” if the second episode of infective endocarditis occurs within 6 months after the initial episode, but is defined as a “recurrence” if the second episode occurs after a period greater than 6 months from the previous episode. However, if a repeat episode of infective endocarditis is shown by molecular analysis to be caused by the same strain, it would be better defined as a “relapse” regardless of the time duration between episodes [1].

## Case presentation

**Fig. 1:**
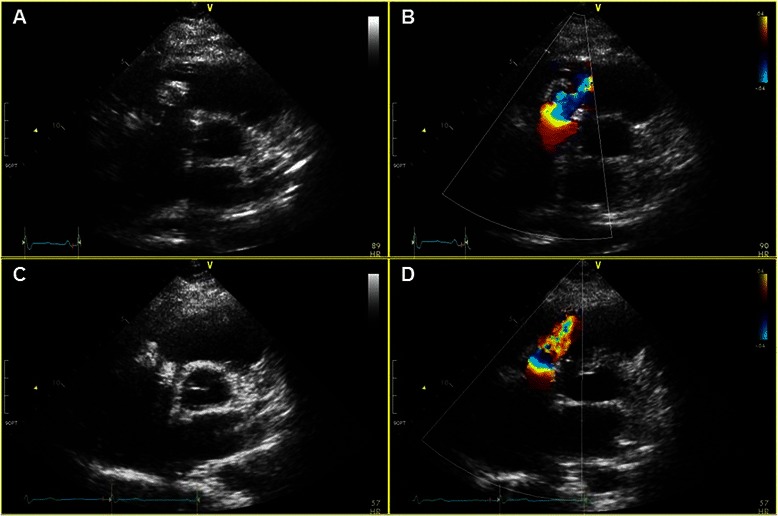
Echocardiograms performed before the patient’s final discharge (**a** and **b**) and 8 months after surgery (**c** and **d**). Both showed a well-functioning aortic valve homograft in the tricuspid position

Eleven years ago, a 27-year-old non-drug abuser woman was hospitalized due to a major burn injuring 55 % of her total body surface area. During her hospital treatment, she developed a sustained fever. Blood cultures revealed multi-drug resistant (MDR) *Pseudomonas aeruginosa* (*P. aeruginosa*) bacteremia. Her fever did not subside in spite of antibiotic therapy, and an echocardiogram showed tricuspid valve vegetation. She was transferred to our hospital and underwent tricuspid valve replacement (TVR) with a porcine bioprosthetic valve (27 mm Hancock II mitral valve; Medtronic, Minneapolis, MN). Her intraoperative valvular tissue cultures also proved to be positive for *P. aeruginosa*. She was treated with carbapenem antibiotics (meropenem) for 6 additional weeks after surgery and her blood cultures after the surgery revealed no bacterial growth. Ten years later, she developed symptoms of fever, nausea, and vomiting and was hospitalized again. MDR *P. aeruginosa* was grown from her blood cultures and her echocardiogram revealed tricuspid valve vegetation with paravalvular leakage. She was initially treated with polymyxin antibiotics (colistimethate sodium; colistin), and underwent redo-TVR with a porcine bioprosthetic valve (25 mm St. Jude Medical Epic valve; St. Jude Medical, St. Paul, MN). Her valvular tissue cultures were negative for *P. aeruginosa*. She received colistin and ciprofloxacin antibiotic therapy for 6 weeks after surgery and her postoperative blood cultures were shown to be negative for bacterial growth. Ten months later, she presented at the hospital again with symptoms of fever and chills. MDR *P. aeruginosa* was identified again by blood culture, and her echocardiogram revealed vegetation on the prosthetic tricuspid valve, with abscess formation in the perivalvular area. Despite 6 weeks of antibiotic therapy with colistin, her blood culture results remained positive for *P. aeruginosa* and her spiking fever persisted. She underwent a second redo-TVR with a bovine bioprosthetic valve (25 mm Carpentier-Edward Perimount; Edwards Lifesciences, Irvine, CA). Tissue cultures from the explanted valve proved to be positive for *P. aeruginosa*, and she was scheduled for treatment with colistin for 6 additional weeks after surgery. However, she developed a fever on postoperative day 39 and her blood culture results again showed MDR *P. aeruginosa* growth. She then was treated with colistin, ciprofloxacin and rifampin; however, her blood cultures were still positive for *P. aeruginosa* and her echocardiogram revealed vegetation on the bioprosthetic valve. Two months after the second redo surgery, she underwent a third redo-TVR with an aortic valve homograft (23 mm) because a mitral valve homograft was not available. Operative findings demonstrated abscessed and necrotic tissues in the septal annular area of the prosthetic valve stent although there was no prosthetic valve dehiscence. Abscessed and necrotic tissues were thoroughly removed. Although a 25 mm sized bioprosthetic valve was used in the previous second redo-TVR, a 23 mm sized aortic valve homograft fitted on the tricuspid annulus well. The aortic valve homograft was tailored appropriately, and the proper location for anchoring the homograft commissures was identified in the right ventricular wall so that the right ventricular outflow tract would align with one of the valve sinuses. The fixation sutures for anchoring the homograft commissures were placed on the septal, anterior, and posteroinferior walls of the right ventricle using interrupted 4-0 polypropylene sutures. The aortic valve homograft was placed in the infra-annular position of the tricuspid annulus, and the tricuspid annulus base was exposed so that the previous abscess site was not concealed by annular sewing sutures. Continuous 4-0 polypropylene suture was placed between the infra-annular right ventricular position of the tricuspid annulus and the homograft aortic valve annulus. Her intraoperative tissue cultures did not reveal any bacterial growth. She received continuous administration of the same antibiotics, and her postoperative blood cultures were negative for bacterial growth. She was discharged from the hospital after 6 additional weeks of antibiotic therapy. The echocardiogram performed before her discharge showed a well-functioning homograft (Fig. 1), and she has been fever-free for more than 12 months at an out-patient clinic.

**Fig. 2:**
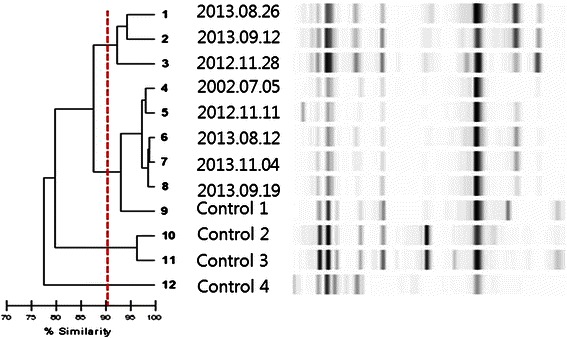
The strain typing by automated repetitive sequence-based polymerase chain reaction analysis of all the pathogens (*P. aeruginosa*) involved in each event of infective endocarditis of the patient. All the pathogens were shown to have similarities greater than 90 %, and therefore were identified as the same strain

In the present case, we identified the strains of each pathogen involved in each event of infective endocarditis by using the automated repetitive sequence-based polymerase chain reaction (rep-PCR) analysis method. This method targets non-coding repetitive sequences which are spread throughout eubacterial genomes [2], and has been shown to be reliable on *P. aeruginosa* isolates [3]. Pathogens from each event of the patient’s infective endocarditis were frozen and stored at −80 °C, and maintained at Microbank (Pro-Lab Diagnostics, Ontario, Canada). *P. aeruginosa* isolates were subcultured on blood agar plates and all bacterial deoxyribonucleic acid (DNA) was extracted using the UltraClean Microbial DNA isolation kit (MO BIO Laboratories, Carlsbad, CA). DNA strain typing was performed using the DiversiLab rep-PCR kit for Pseudomonas fingerprinting (bioMérieux, Durham, NC) (Fig. 2). The results showed similarities of 90 % to 95 % in all strains. Although 95 % or greater similarity is generally accepted as the same strain [3], it seems the result is sufficient for identification as the same strain in all samples considering epidemiologic circumstances in this case.

## Discussion

Patients who have recovered from infective endocarditis remain at risk for an additional episode of infective endocarditis, and the lifetime risk of a second episode of infective endocarditis has been estimated to be between 2 % and 22 % [4–8]. Repeat infective endocarditis due to the same species is traditionally defined as a “relapse” if the second episode of infective endocarditis occurs within 6 months after the initial episode, but is defined as a “recurrence” if the second episode occurs after a period greater than 6 months from the previous episode. However, if a repeat episode of infective endocarditis is shown by molecular analysis to be caused by the same strain, it would be better defined as a “relapse” regardless of the time duration between episodes [1].

In the present case, a non-drug abuser patient suffered from three repeat attacks of infective endocarditis caused by the same pathogen, MDR *P. aeruginosa*, 10 years, 10 months, and 2 months after previous surgery, respectively. The mechanism for repeat infective endocarditis with the same pathogen can be categorized as reactivation of a chronic silent infection (relapse), or a new episode of infection by a different microorganism (reinfection or recurrence). In the present case, we tried to elucidate the serial episodes of infection by genotyping the bacteria. Based on the automated rep-PCR DNA strain typing results, all the pathogens were identified as the same strain. The patient therefore was regarded as having multiple events of relapsing infective endocarditis, although there is a hypothetical possibility of reinfection with a persistently colonizing strain.

Because repeated attacks of infective endocarditis occurred due to the same strain of a pathogen which is resistant to multiple antibiotics, we tried to prevent further recurrence. First, we used colistin as a main antibiotic therapy because it has been proven to have acceptable effectiveness on MDR *P. aeruginosa* [9]. Second, we tried to remove all abscessed and necrotic tissues intraoperatively and exposed the tricuspid annulus base so that the previous abscess site was not concealed by annular sewing sutures. Third, we used an aortic homograft for the third redo-TVR. Completely extirpating the infected tissue and avoiding placement of any prosthetic material could have been considered because the previous TVRs with three different bioprosthetic valves all failed to treat recurrent infective endocarditis. However, we decided to use a homograft because the extirpation procedure could lead to late-onset right-sided failure in this young woman, and valve replacement using a homograft is known to be more resistant to recurrent endocarditis [10].

## Conclusions

Based on the automated rep-PCR DNA strain typing results, all the pathogens in 11 years were identified as the same strain. The patient therefore can be regarded as having multiple events of relapsing infective endocarditis caused by same strain of *P. aeruginosa*. This is the first case report of tricuspid valve replacement using an aortic valve homograft to treat relapsing endocarditis.

## Consent

Written informed consent was obtained from the patient for publication of this Case report and any accompanying images. A copy of the written consent is available for review by the Editor-in-Chief of this journal.

## Abbreviations

MDR: Multi-drug resistant
*P. aeruginosa*: *Pseudomonas aeruginosa*
TVR: Tricuspid valve replacement
rep-PCR: repetitive sequence-based polymerase chain reaction
DNA: Deoxyribonucleic acid

## References

[1] Chu VH, Sexton DJ, Cabell CH, Reller LB, Pappas PA, Singh RK, Fowler VG, Corey GR, Aksoy O, Woods CW (2005). Repeat infective endocarditis: Differentiating relapse from reinfection. Clin Infect Dis.

[2] Healy M, Huong J, Bittner T, Lising M, Frye S, Raza S, Schrock R, Manry J, Renwick A, Nieto R, Woods C, Versalovic J, Lupski JR (2005). Microbial DNA typing by automated repetitive-sequence-based PCR. J Clin Microbiol.

[3] Doleans-Jordheim A, Cournoyer B, Bergeron E, Croize J, Salord H, Andre J, Mazoyer M-A, Renaud FNR, Freney J (2009). Reliability of Pseudomonas aeruginosa semi-automated rep-PCR genotyping in various epidemiological situations. Eur J Clin Microbiol Infect Dis.

[4] Tornos MP, Permanyer-Miralda G, Olona M, Gil M, Galve E, Almirante B, Soler-Soler J (1992). Long-term complications of native valve infective endocarditis in non-addicts. A 15-year follow-up study. Ann Intern Med.

[5] Castillo JC, Anguita MP, Ramirez A, Siles JR, Torres F, Mesa D, Franco M, Munoz I, Concha M, Valles F (2000). Long term outcome of infective endocarditis in patients who were not drug addicts : a 10 year study. Heart.

[6] Mansur AJ, Dal Bo CMR, Fukushima JT, Issa VS, Grinberg M, Pomerantzeff PMA (2001). Relapses, recurrences, valve replacements, and mortality during the long-term follow-up after infective endocarditis. Am Heart J.

[7] Renzulli A, Carozza A, Romano G, De Feo M, Della Corte A, Gregorio R, Cotrufo M (2001). Recurrent infective endocarditis : A multivariate analysis of 21 years of experience. Ann Thorac Surg.

[8] Mylonakis E, Calderwood SB (2001). Infective endocarditis in adults. N Engl J Med.

[9] Matthew EF, Sofia KK (2005). Colistin: The revival of polymyxins for the management of multidrug-resistant gram-negative bacterial infections. Clin Infect Dis.

[10] Niwaya K, Knott-Craig CJ, Santangelo K, Lane MM, Chandrasekaran K, Elkins RC (1999). Advantage of autograft and homograft valve replacement for complex aortic valve endocarditis. Ann Thorac Surg.

